# Synthesis, Antioxidant and Antiproliferative Actions of 4-(1,2,3-Triazol-1-yl)quinolin-2(1*H*)-ones as Multi-Target Inhibitors

**DOI:** 10.3390/ijms241713300

**Published:** 2023-08-27

**Authors:** Essmat M. El-Sheref, Stefan Bräse, Hendawy N. Tawfeek, Fatmah Ali Alasmary, Bahaa G. M. Youssif

**Affiliations:** 1 Chemistry Department, Faculty of Science, Minia University, El-Minia 61519, Egypt; hendawy1976@yahoo.com; 2 Institute of Biological and Chemical Systems, IBCS-FMS, Karlsruhe Institute of Technology, 76131 Karlsruhe, Germany; 3 Unit of Occupational of Safety and Health, Administration Office of Minia University, El-Minia 61519, Egypt; 4 Department of Chemistry, College of Science, King Saud University, Riyadh 11451, Saudi Arabia; fasmari@ksu.edu.sa; 5 Pharmaceutical Organic Chemistry Department, Faculty of Pharmacy, Assiut University, Assiut 71526, Egypt; bgyoussif2@gmail.com

**Keywords:** quinoline, triazole, antiproliferative, antioxidant, apoptosis

## Abstract

The reaction of 4-azido-quinolin-2(1*H*)-ones **1a**–**e** with the active methylene compounds pentane-2,4-dione (**2a**), 1,3-diphenylpropane-1,3-dione (**2b**), and K_2_CO_3_ was investigated in this study. This approach afforded 4-(1,2,3-triazol-1-yl)quinolin-2(1*H*)-ones **3a**–**j** in high yields and purity. All newly synthesized products’ structures were identified. Compounds **3a**–**j** were tested for antiproliferative activity against a panel of four cancer cell lines. In comparison to the reference erlotinib (GI_50_ = 33), compounds **3f**–**j** were the most potent derivatives, with GI_50_ values ranging from 22 nM to 31 nM. The most effective antiproliferative derivatives, **3f**–**j**, were subsequently investigated as possible multi-target inhibitors of EGFR, BRAF^V600E^, and EGFR^T790M^. Compound **3h** was the most potent inhibitor of the studied molecular targets, with IC_50_ values of 57 nM, 68 nM, and 9.70 nM, respectively. The apoptotic assay results demonstrated that compounds **3g** and **3h** function as caspase-3, 8, and Bax activators as well as down-regulators of the antiapoptotic Bcl2, and hence can be classified as apoptotic inducers. Finally, compounds **3g** and **3h** displayed promising antioxidant activity at 10 µM, with DPPH radical scavenging of 70.6% and 73.5%, respectively, compared to Trolox (77.6%).

## 1. Introduction

Despite scientific and social advancements, cancer remains one of the most prevalent diseases of concern and a primary cause of human suffering. Cancer deaths globally are expected to climb by more than 13.1 million by 2030, according to estimates [1,2,3,4]. As a result, the development of newer and more potent anticancer treatments with stronger selectivity on neoplastic cells and fewer side effects, capable of overcoming challenges such as extreme toxicity and resistance to existing drugs, may be contemplated [5,6,7]. The potential that some compounds with antioxidant properties can explain chemopreventive action is a matter of ongoing discussion. Indeed, numerous previous studies have found that antioxidants can improve existing chemotherapy protocols by reducing hazardous side effects while maintaining treatment efficacy [8,9,10]. Furthermore, further in vitro studies suggest that these compounds play an important role in causing apoptosis in cancer cells [11,12].

Cancer is a term used to describe a group of diseases caused by abnormalities in cell proliferation and replication [13]. Cancer cells typically have a large number of mutations; no two samples from the same patient are same [14,15,16]. As a result, only medications that act on many cancer-related pathways at the same time can achieve improved drug efficacy and minimize the risk of drug resistance. Multi-targeted medications have long been used in the clinic in the forms of both “Cocktail Therapy” [17,18], which combines numerous drugs, and “multi-component drugs” [19,20], which combine two or more drugs in a single tablet. A combination of targeted medicines has been authorized as an effective strategy to cure cancer. A pharmacogenomic platform was developed for the quick identification of drug combinations that can overcome resistance in specific individuals [21,22].

Although these strategies can achieve poly-pharmacological effects, they have encountered unavoidable challenges, such as the difficulty and length of time required to determine the best therapeutic pairings and timing, the difficulty in managing the bio-distribution characteristics and pharmacokinetics of a particular treatment, potential interactions between several drugs that lead to potential side effects, and low patient tolerance [23,24]. Alternatively, the development of a single chemical entity containing a pharmacological combination that works on many cancer-relevant sites could potentially overcome these issues. These anticancer drugs, known as “single molecule multiple targets”, “multiple ligands”, or “hybrids”, have received a lot of interest in recent years [25,26,27,28,29]. Multiple ligands provide certain distinct advantages over cocktail and multi-component medications, including reduced risk of drug interactions, simplified drug metabolism, enhanced drug transport, and lower drug research and development expenses. These characteristics make them promising candidates for the development of the next generation of anticancer medicines. In reality, some medications currently in clinical trials target many ligands, while this was not their intention at the outset. For example, the FDA-approved kinase inhibitors sorafenib [30] and sunitinib [31], which are utilized in the clinic for cancer therapy, target different types of kinases.

Several antineoplastic drugs with various structures have been developed as a consequence of research efforts over the last few decades [32,33]. Due to its biodiversity and plasticity, the quinoline nucleus has been a highly favored motif for the target-based design and development of anticancer agents [34]. Quinolone derivatives, on the other hand, have an important place in medicinal chemistry due to their distinct structure and recognized therapeutic impact; whether natural or synthetic, they have demonstrated a wide range of pharmacological actions [35,36], with a promising role in the improvement of anti-cancer drug resistance [37,38,39]. Furthermore, several quinolones have been discovered to have great effects in a variety of operations, including growth inhibition by cell cycle arrest, apoptosis, and angiogenesis inhibition [40,41,42].

**Figure 1:**
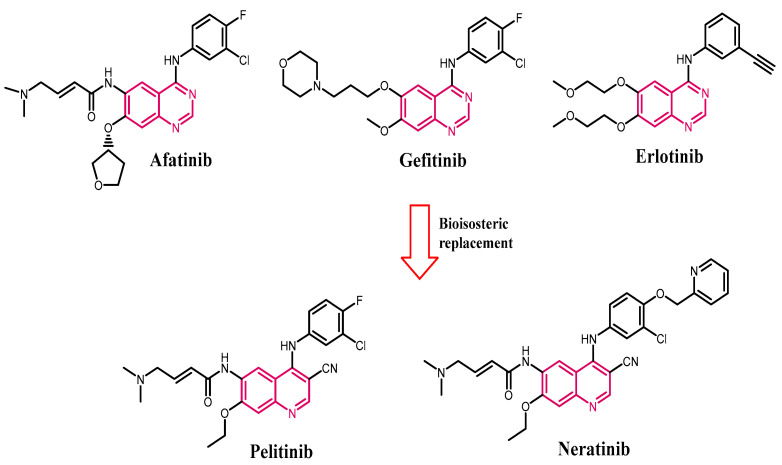
Structures of some reported EGFR inhibitors.

Moreover, small compounds, such as quinazoline derivatives, can suppress EGFR by inhibiting tyrosine kinase at ATP-binding sites [43,44]. The FDA has approved afatinib, gefitinib, and erlotinib (Figure 1), quinazoline derivatives designed to block EGFR kinase, for the treatment of non-small-cell lung and breast cancers [45,46]. The bioisosteric replacement of the quinazoline ring system with quinoline resulted in quinoline derivatives such as neratinib and pelitinib (Figure 1), which are potent EGFR kinase inhibitors [47,48]. Many researchers were encouraged by the above-mentioned results to design and synthesize a series of quinoline-based EGFR inhibitors, which are expected to be as potent as structurally related quinazoline bioisosteres.

**Figure 2:**
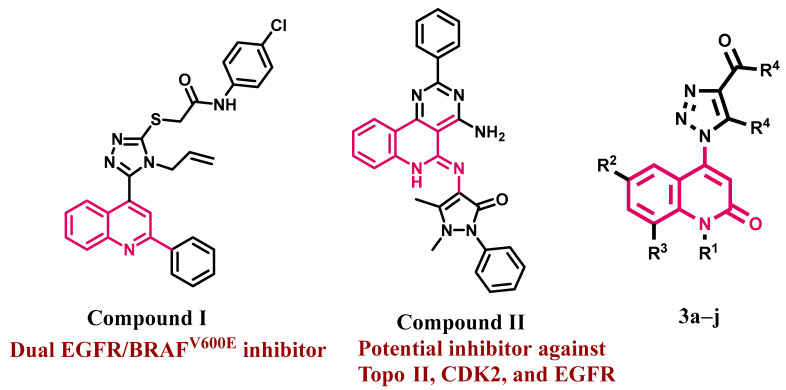
Structures of compounds **I** and **II** and newly synthesized **3a**–**j**.

In a recent publication [34], we described the design, synthesis, and antiproliferative activity of a novel class of quinoline-based compounds as possible dual inhibitors of EGFR and BRAF^V600E^. Compound **I** (Figure 2) was the most effective derivative, with a GI_50_ value of 3.30 µM against four cancer cell lines, when compared to the reference doxorubicin (GI_50_ = 1.13 µM). Compound **I** inhibited both EGFR and BRAF^V600E^ with IC_50_ values of 1.30 µM and 3.80 µM, respectively. Another study [49] described the development of a novel set of quinoline-based compounds as possible multi-target antiproliferative agents. Compound **II** (Figure 2) was proven to be the most potent derivative, with a GI_50_ value of 1.05 µM against the four cancer cell lines examined, when compared to doxorubicin (GI_50_ = 1.10 µM). Compound **II** demonstrated the best topo II inhibitory activity at the examined concentrations (100 µM = 47.6% and 20 µM = 19.5%) when compared to the positive control, etoposide (100 µM = 83.7% and 20 µM = 66.0%). Moreover, compound **II** exhibited promising dual inhibitory action against CDK2 and EGFR with IC_50_ values of 1.60 µM and 0.40 µM, respectively.

Additionally, 1,2,3-triazoles have found substantial uses in pharmaceutical chemistry [50,51,52,53] due to their ease of synthesis, and the click procedure was the best and most common way to synthesize 1,2,3-triazole ring via copper(I)-catalyzed azide–alkyne cycloaddition [54,55,56,57]. Because of their potential biological actions, scientific attention is now focused on the synthesis of 1,4- and 1,5-disubstituted 1,2,3-triazoles [52,58].

Motivated by these findings, and in the pursuit of a new antiproliferative agent with potential multi-target inhibitory action [27,28,29,59,60,61,62], we present here the design, synthesis, and antiproliferative activity of a new series of quinoline/1,2,3-triazole hybrids **3a**–**j** (Figure 2) as potential multi-target inhibitors.

Compounds **3a**–**j** were evaluated for cell viability against a normal human cell line (human mammary gland epithelial (MCF-10A) cell line). All of the newly synthesized analogues were tested for antiproliferative activity against four cancer cell lines. The most potent derivatives were studied further as multi-target inhibitors against wild-type EGFR, mutant-type EGFR (EGFR^T790M^), and BRAF^V600E^. The most active derivative’s potential apoptotic and antioxidant capabilities were also examined.

## 2. Results and Discussion

### 2.1. Chemistry

**Scheme 1:**
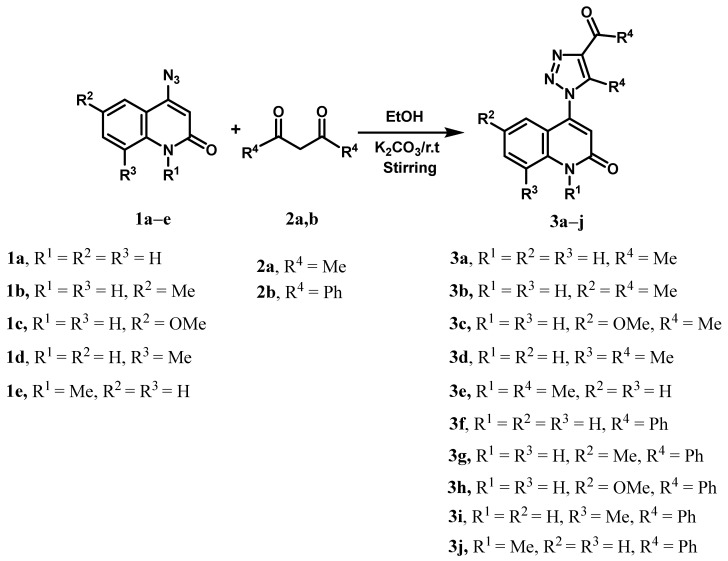
Synthesis of 4-(1,2,3-triazol-1-yl)quinolin-2-ones (**3a**–**j**).

Previously, our research team has identified numerous quinolone derivatives with a 1,2,3-triazole ring. In addition, in recent publications, we described an effective methodology for the synthesis of 4-(1,2,3-triazol-2-yl)quninolin-2-ones via the click reaction and investigated their biological characteristics [63,64]. However, in this study, we synthesize analogous 1,2,3-triazoloquinolone derivatives by reacting 4-azido-2-quinolinones **1a**–**e** with active methylene compounds such as pentane-2,4-dione (**2a**) and 1,3-diphenylpropane-1,3-dione (**2b**) (Scheme 1).

To optimize the reaction condition, we performed the reaction by starting with 4-azidoquinolin-2(1*H*)-one (**1a**) and pentane-2,4-dione (**2a**) as active methylene compounds and various bases at room temperature, worming, and under refluxing temperature. When we employed other bases to optimize our conditions, such as Et3N, piperidin, NaOH, and KOH in ethanol as a solvent, we obtained compound **3a** with a negligible yield of less than 20% when either cold or hot (Table 1). By contrast, in the presence of inorganic bases, namely K_2_CO_3_ in ethanol, product **3a** was obtained in excellent yields of 90%. By performing the reaction under the above optimizing conditions with 1,3-diphenylpropane-1,3-dione (**2b**), we obtained the product **3f** in an excellent yield as 82%. So, the best result was obtained when the reaction was conducted by starting with 4-azidoquinolin-2(1*H*)-one **1a**–**e** (1 mmol), the active methylene compounds **2a**,**b** (1 mmol), and 1.2 mmol of K_2_CO_3_ in ethanol according to a previous publication [65].

The structures for all obtained products are fully consistent with their spectral data, such as ^1^H NMR, ^13^C NMR spectrum, mass spectrometry, and elemental analysis. To confirm our results, we choose compound **3b**, which was assigned as 4-(4-acetyl-5-methyl-1*H*-1,2,3-triazol-1-yl)-6-methylquinolin-2(1*H*)-one (Figure 1). Mass spectrometry and elemental analysis for compound **3b** indicate that it has the molecular formula of C_15_H_14_N_4_O_2_ with *m/z* 282, which indicate that it is the result of a combination between one molecule of 4-azido-6-methylquinolin-2(1*H*)-ones (**1b**) and one molecule of acetyl acetone (**2a**) with the elimination of one molecule of H_2_O. The ^1^H NMR spectra for compound **3b** showed five singlet signals in a ratio (1:1:3:3:3) with chemical shifts at δ_H_ 12.26, 7.40, 6.87, 3.32, 2.45, and 2.26 ppm, which were assigned as NH, H-5, COCH_3_, CH_3_ (H-5a’), and CH_3_ (H-6a), respectively. The three methyl groups were further confirmed from their ^13^C NMR spectra, which gave signals at δ_C_ 27.67 (COCH_3_), 20.38 (CH_3_-6a), and 9.29 ppm (CH_3_-5a’).

**Figure 3:**
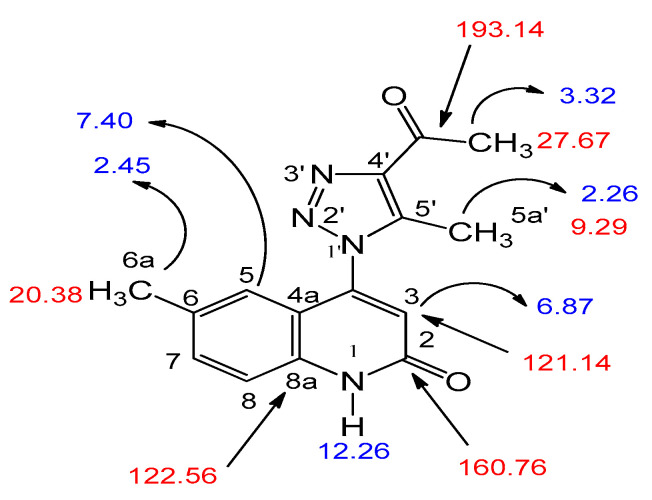
The structure elucidation for compound **3b**.

Also, the ^13^C NMR spectra clearly showed the presence of two downfield signals at δ_C_ 193.14 and 160.76 ppm, which were assigned as (COCH_3_) and quniolinone-C-2, respectively, as shown in Figure 3. All spectral signals that appeared in ^1^H NMR spectra or in ^13^C NMR spectra were identical to those in mass spectrometry and elemental analysis, and to the structure, which was deduced exactly.

Furthermore, in all obtained products **3a**–**j**, the elemental analysis and mass spectrometry clearly show that the gross formula for all compounds came from the reaction between one mole of 4-azidoquinolin-2(1*H*)-ones (**1a**–**e**) and one mole of the active methylene (**2a**,**b**) with the elimination of one H_2_O molecule. Also, the ^1^H NMR clearly showed the presence of a downfield singlet signal at δ_H_ = 6.86–6.96 ppm, which was assigned as quinolinon-H-3 and confirmed with ^13^C NMR with a chemical shift at δ_C_ = 120.38–122.24 ppm for all obtained products. Additionally, the presence of two carbonyl groups was confirmed by the ^13^C NMR with a chemical shift at δ_C_ = 160.20–160.97, 186.08–186.18, and 193.09–193.18 ppm, for all quinolinon-C-2 (for compounds **3a**–**j**), COCH_3_ (for compounds **3a**–**e**), and COPh (compounds **3f**–**j**), respectively.

Finally, through the chemical shift’s value of the external carbonyl group COR as shown above, which refers to the nature of the group attached to the carbonyl, as the methyl group is a donor group and the phenyl group is withdrawn, the first appears at a lower value in the ^13^C NMR spectroscopy. Through the preceding values of the different analyses, the validity of the chemical composition of all the compounds that were obtained is clear, and it is clear that the behavior of the 4-azidoquinoli-2(1*H*)-ones **1a**–**e** with the active methylene **2a**,**b**, whether aromatic or aliphatic, exhibited the same behavior during the reaction as shown in Scheme 1.

**Scheme 2:**
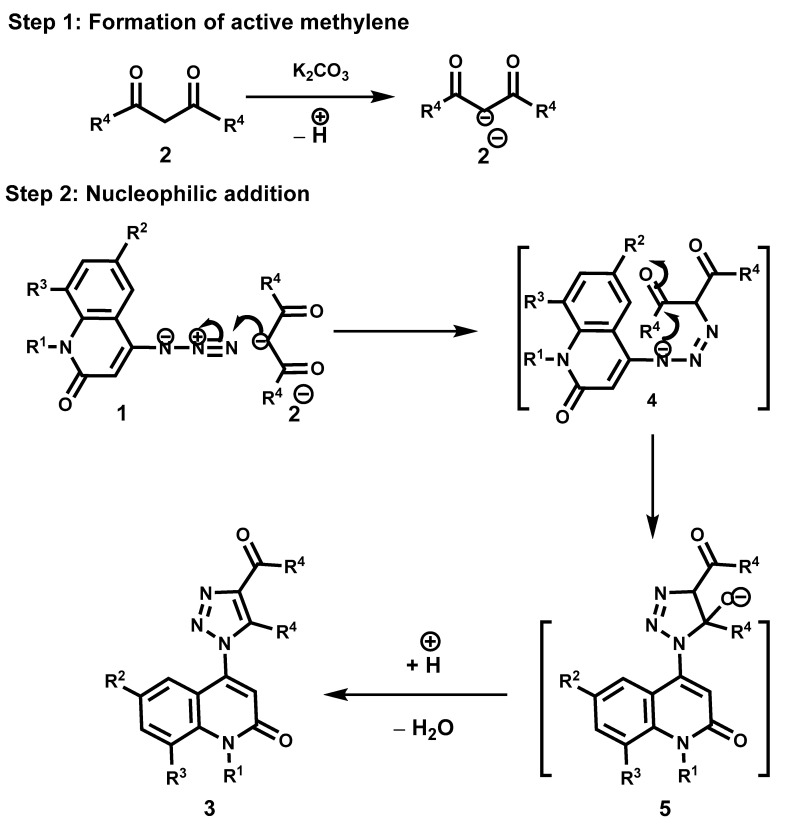
Suggested mechanism for the formation of compounds **3a**–**j.**

The formation of compounds **3a**–**j** can be rationalized with the following suggested mechanism (Scheme 2) via the two steps shown below. The first step included the formation of active methylene via abstracting a hydrogen proton from **2a**,**b** by K_2_CO_3_. The second step involved 1,3-dipolar cycloaddition of the enolate **2^−^** on the *N3*-group of the 4-azidoquinolin-2(1*H*)-ones **1a**–**e** and forming the intermediate **4**, and then intra-nucleophilic addition from the *N*-3 atom of the **1a**–**e** on the carbonyl group and cyclization resulting in the adduct **5** which accepted a hydrogen proton and the loss of a H_2_O molecule to give the final products **3a**–**j**.

### 2.2. Biochemical Assays

#### 2.2.1. Cell Viability Assay

The human mammary gland epithelial (MCF-10A) cell line was used to investigate the viability of the 1,2,3-triazole-based derivatives **3a**–**j**. Compounds **3a**–**j** were cultured on MCF-10A cells for four days before being evaluated for vitality using the MTT assay [66,67]. According to Table 1, none of the compounds examined displayed cytotoxic effects, and the cell viability for the compounds tested at 50 µM was greater than 87%.

#### 2.2.2. Antiproliferative Assay

The antiproliferative activity of **3a**–**j** was evaluated against four human cancer cell lines using the MTT assay [49,68] and erlotinib as the reference drug: Panc-1 (pancreatic cancer cell line), MCF-7 (breast cancer cell line), HT-29 (colon cancer cell line), and A-549 (human epithelial cancer cell line). Table 2 displays the median inhibitory concentration (IC_50_) and the average of IC_50_ (GI_50_).

In general, compounds **3a**–**j** had promising antiproliferative activity, with GI_50_ against the four cancer cell lines tested ranging from 22 nM to 65 nM, compared to erlotinib’s 33 nM. The newly synthesized compounds **3a**–**j** can be divided into two major Scaffolds. Scaffold A compounds are 4-acetly, 5-methyl-1,2,3-triazoles **3a**–**e** (R_4_ = CH_3_), and scaffold B compounds are 4-benzoyl, 5-phenyl-1,2,3-triazoles **3f**–**j** (R_4_ = Ph). Compounds **3a**–**e** (R^4^ = CH_3_) showed GI_50_ values ranging from 39 nM to 65 nM and were found to be less potent than congeners **3f**–**j** (R^4^ = Ph) of GI_50_ values ranging from 22 nM to 31 nM, indicating the relevance of the phenyl group of 1,2,3-triazole moiety for the antiproliferative action.

Compounds **3f**, **3g**, **3h**, and **3i** (Scaffold B) were revealed to be the most potent derivatives, with GI_50_ values of 28, 26, 22, and 31 nM, respectively. These compounds outperformed the reference drug erlotinib (GI_50_ = 33 nM). Compounds **3f**, **3g**, and **3h** were found to be more effective than erlotinib against all cancer cell lines tested.

Compound **3h** (R^1^ = R^3^ = H, R^2^ = OCH_3_, R^4^ = Ph) was the most potent derivative among all synthesized derivatives, with a GI_50_ value of 22 nM, 1.5-fold more potent than the reference erlotinib. The phenyl ring substitution on the 1,2,3-triazole moiety results in compound **3c** (R^1^ = R^3^ = H, R^2^ = OCH_3_, R^4^ = CH_3_), which had a GI_50_ value of 39 nM and was 1.8-fold less potent than compound **3h**, indicating the relevance of the phenyl ring substitution on the 1,2,3-triazole moiety.

Another significant point to consider is the relevance of the substitution pattern at position five on the quinoline moiety, as seen in compounds **3g** (R^1^ = R^3^ = H, R^2^ = CH_3_, R^4^ = Ph) and **3f** (R^1^ = R^3^ = H, R^2^ = H, R^4^ = Ph). Compounds **3g** and **3f** have GI_50_ values of 26 nM and 28 nM, respectively, and are both less potent than **3h** (R^1^ = R^3^ = H, R^2^ = OCH_3_, R^4^ = Ph), demonstrating the importance of the substitution pattern of position five of the quinoline moiety on the antiproliferative activity of the newly synthesized compounds and that the activity increases in the order OCH_3_ > CH_3_ > H. Additionally, compound **3i** (R^1^ = CH_3_, R^3^ = H, R^2^ = H, R^4^ = Ph) had a GI_50_ value of 35 nM, which was less potent than compound **3f** (R^1^ = R^3^ = H, R^2^ = H, R^4^ = Ph), suggesting the relevance of the quinoline moiety’s Free N-1 atom for antiproliferative action.

Finally, as stated above, Scaffold A compounds **3a**–**e** (R_4_ = CH_3_) were less potent than compounds **3f**–**j** (R^4^ = Ph), with GI_50_ values ranging from 39 nM to 65 nM, where all parameters that affect the activity of compounds **3f**–**j** were also present in compounds **3a**–**e**. For example, compound **3c** (R^1^ = R^3^ = H, R^2^ = OCH_3_, R^4^ = CH_3_) had a GI_50_ value of 39 nM against the four cancer cell lines tested, being more potent than compounds **3b** (R^1^ = R^3^ = H, R^2^ = CH_3_, R^4^ = Ph) and **3a** (R^1^ = R^3^ = H, R^2^ = H, R^4^ = Ph), indicating the relevance of the methoxy group at the fifth position of the quinoline ring to antiproliferative action. Moreover, compound **3d** (R^1^ = CH_3_, R^3^ = H, R^2^ = H, R^4^ = CH_3_) had a GI_50_ value of 62 nM, being 1.4-fold less potent than **3a**, indicating the relevance of the quinoline moiety’s Free N-1 atom for antiproliferative action.

#### 2.2.3. EGFR Inhibitory Assay

**Figure 4:**
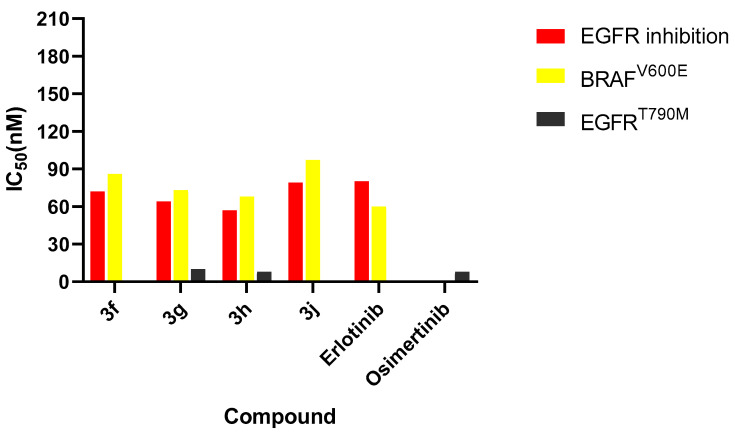
IC_50_ of compounds **3f**, **3g**, **3h**, and **3j** against EGFR, EGFR^T790M^, and BRAF^V600E^.

Compounds **3f**, **3g**, **3h**, and **3j**, the most effective antiproliferative derivatives, were evaluated for their inhibitory action on EGFR as a potential target for their antiproliferative action [69]. Table 3 and Figure 4 show the results as IC_50_ values versus erlotinib as a reference drug. The investigated compounds **3f**, **3g**, **3h**, and **3j** demonstrated promising EGFR inhibitory action with IC_50_ values of 72 nM, 64 nM, 57 nM, and 79 nM, respectively. The results of the EGFR inhibitory assay corroborate the results of the antiproliferative assay, with the most active antiproliferative derivatives also being the most active EGFR inhibitors, showing that EGFR may be a viable target for antiproliferative activity. Compounds **3f**, **3g**, and **3h** demonstrated superior EGFR inhibitory action to the reference erlotinib, where compound **3j** (IC_50_ = 79 nM) was equipotent to erlotinib (IC_50_ = 80 nM). The most potent antiproliferative agent, compound **3h**, was also the most potent EGFR inhibitor, with an IC_50_ value of 57 nM, being 1.4-fold more potent than erlotinib.

#### 2.2.4. BRAF^V600E^ Inhibitory Assay

Compounds **3f**, **3g**, **3h**, and **3j** were tested for their ability to inhibit mutant BRAF [62]. Table 3 and Figure 4 display the results as IC_50_ values. With IC_50_ values ranging from 68 nM to 93 nM, the compounds examined showed potential inhibitory activity against BRAF^V600E^. In all cases, the investigated derivatives were shown to be less effective as BRAF^V600E^ inhibitors than erlotinib (IC_50_ = 60 ± 5 nM). The most potent antiproliferative agent and EGFR inhibitor, compound **3h**, was also the most potent BRAF^V600E^ inhibitor, with an IC_50_ value of 68 ± 5 nM, comparable to erlotinib in BRAF^V600E^ inhibitory action. Compounds **3f** and **3g** were shown to be the second and third most active, with IC_50_ values of 86 ± 6 nM and 73 ± 6 nM, respectively. Finally, compound **3j** was the least effective BRAF^V600E^ inhibitor, with an IC_50_ of 97 ± 7 nM, being 1.6-fold less potent than the reference erlotinib (IC_50_ = 60 ± 5 nM). Compounds **3g** and **3h** were discovered to be potential antiproliferative agents with dual EGFR and BRAF^V600E^ inhibitory action, requiring significant structural modifications in their backbone structures to optimize their biological activity.

#### 2.2.5. EGFR^T790M^ Inhibitory Assay

Compounds **3g** and **3h**’s encouraging results as antiproliferative agents with potential EGFR and BRAF^V600E^ inhibitory activities motivated us to test their efficacy on the mutant EGFR (EGFR ^T790M^) receptor type [59]. Results were cited as IC_50_ values in Table 3 and Figure 4 against osimertinib as a reference drug. Compounds **3g** and **3h** inhibited EGFR^T790M^ with IC_50_ values of 8.40 ± 0.7 nM and 9.70 ± 0.8 nM, which were equivalent to the reference Osimertinib’s IC_50_ value of 8 nM. These findings add to the evidence of the investigated compounds’ antiproliferative activity, which can serve as multi-target inhibitors.

#### 2.2.6. Apoptotic Assays

Controlling or perhaps terminating the uncontrolled proliferation of cancer cells is one method of treating cancer. Using the cell’s natural dying process is a very successful strategy. Apoptosis evasion is a hallmark of cancer and is not particular to the cause or kind of cancer, and hence targeting apoptosis is useful for many types of cancer. Many anticancer medications target different phases in both the intrinsic and extrinsic pathways [70,71,72]. Compounds **3f**, **3g**, and **3h** were tested for their capacity to activate the apoptosis cascade and disclose their proapoptotic potential.

##### Caspase-3 Assay

Caspases play a crucial function in the induction and achievement of apoptosis. Caspase-3 is an essential caspase that cleaves different proteins in cells, resulting in apoptosis [73,74]. The most potent derivatives in all in vitro studies, compounds **3f**, **3g**, and **3h**, were tested as caspase-3 activators against the human epithelial cancer cell line (A-594) [29], and the findings are reported in Table 4. The results showed that compounds **3f**, **3g**, and **3h** had promising caspase-3 protein overexpression levels of 524 ± 5, 587 ± 5, and 715 ± 6 pg/mL, respectively. They elevated the protein caspase-3 in the A-594 cancer cell line by approximately 8, 9, and 11 times when compared to untreated control cells. In all cases, the investigated compounds **3g**, **3f**, and **3h** were shown to be more active than the standard staurosporine, which had a caspase-3 level overexpression of 465 ± 4 pg/mL. These findings demonstrated the investigated compounds’ apoptotic potential, which may explain their antiproliferative activity.

##### Caspase 8, Bax, and Bcl-2 Level Assays

Compounds **3g** and **3h** were investigated further for their effect on caspase-8, Bax, and antiapoptotic Bacl-2 levels against the human epithelial cancer cell line (A-594) using staurosporine as a control, as shown in Table 4. Compared to staurosporine, **3g** and **3h** noticeably increased caspase-8 and Bax levels.

Caspase-8 overexpression was highest in compound **3h** (2.35 ng/mL), followed by **3g** (2.17 ng/mL) and standard staurosporine (1.85 ng/mL). In comparison to the control untreated cells, **3g** and **3h** increased caspase-8 levels by 24-fold and 26-fold, respectively.

Moreover, when compared to untreated A-594 cancer cells, compounds **3g** and **3h** induced Bax 36- and 37-fold greater (315 pg/mL and 336 pg/mL, respectively) than staurosporine (288 pg/mL, a 32-fold induction). Finally, compared to staurosporine, compounds **3g** and **3g** elicited equipotent down-regulation of anti-apoptotic Bcl-2 protein levels in the A-594 cell line. These findings suggest that **3g** and **3h** function as caspase-3, 8, and Bax activators and down-regulators of the antiapoptotic Bcl2, and hence can be classified as apoptotic inducers.

#### 2.2.7. Antioxidant Activity

Antioxidant compounds have assumed a key position in medicine because of their widespread preventive and therapeutic application in various disorders. Free radicals play an essential part in cancer, cardiovascular and auto-immune disorders, and aging-related problems, leading to new medical approaches [61]. The scavenging of stable free radicals by 2,2-diphenyl-1-picrylhydrazyl (DPPH) was used to investigate the potential antioxidant properties of compounds **3f**, **3g**, and **3h**, using Trolox as a control (Table 5) [75].

The assay was carried out at three different concentrations of the investigated compounds (100 µM, 50 µM, and 10 µM). Compounds **3g** and **3h** displayed promising antioxidant activity at 10 µM, with DPPH radical scavenging of 70.6% and 73.5%, respectively, compared to Trolox (77.6%). Compounds **3g** and **3h** show comparable radical scavenging activity to Trolox at 100 and 50 µM, respectively (Table 4). Compound **3f** was determined to be the least active compound regarding antioxidant activity. These findings indicated that compounds **3g** and **3h** could be regarded as potent antiproliferative agents with antioxidant action.

## 3. Materials and Methods

### 3.1. Chemistry

**General information:** refer to Additional information (Supplementary File Figures S1–S32)

Starting materials

4-azidoquinolin-2(1*H*)-ones **1a**–**e** were prepared according to the published literature [76,77]. The active methylene compounds used were pentane-2,4-dione (**2a**) and 1,3-diphenylpropane-1,3-dione (**2b**), and they were used as purchased (Merck).

**General procedure for the synthesis of 4-(1,2,3-triazol-1-yl)quinolin-2-ones (3a–j)** [78,79]

In 30 mL of ethanol, we combined 4-azido-2-quinolinones **1a**–**e** (1 mmol), active methylene ketones **2a**, **b** (1.0 mmol), and K_2_CO_3_ (1.2 mmol). For 20 h, the reaction mixture was stirred at room temperature. Compounds **3a**–**j** were obtained after the reaction was completed by pouring them into a 500 mL beaker containing 200 gm of crushed ice (in the case of the reaction with **2b**) or by evaporating the solvent (in the case of the reaction with **2a**). All of the obtained **3a**–**j** products were recrystallized from ethanol.

#### 3.1.1. 4-(4-Acetyl-5-methyl-1*H*-1,2,3-triazol-1-yl)quinolin-2(1*H*)-one (**3a**)

This compound was found to be a pale yellow solid; mp 243-45 °C; yield 90%; ^1^H NMR (DMSO-*d_6_*): *δ*_H_ 12.28 (s, 1H, NH), 7.63 (t, 1H, *J* = 7.5 Hz, H-7), 7.50 (d, 1H, *J* = 8.4 Hz, H-8), 7.18 (t, *J* = 7.8 Hz, 1H, H-6), 6.90 (d, 1H, *J* = 9.3 Hz, H-5), 6.89 (s, 1H, H-3), 3.32 (s, 3H, COCH_3_), 2.35 ppm (s, 3H, H-5a’); ^13^C NMR (DMSO-*d_6_*): *δ*_C_ 193.18 (CO), 160.97 (CO, C-2), 142.60 (C-4), 142.24 (C-5’), 139.39 (C-4’), 139.24 (C-7), 132.15 (C-5), 123.46 (C-8a), 121.26 (C-6), 120.68 (C-3), 115.98 (C-8), 115.57 (C-4a), 27.74 (CH_3_), 9.31 ppm (CH_3_); EI-MS: *m/z* 268 (M^+^, 17). *Anal. Calcd for* C_14_H_12_N_4_O_2_: C, 62.68; H, 4.51; N, 20.88. Found: C, 62.77; H, 4.39; N, 20.69.

#### 3.1.2. 4-(4-Acetyl-5-methyl-1*H*-1,2,3-triazol-1-yl)-6-methylquinolin-2(1*H*)-one (**3b**)

This compound was found to be a pale yellow solid; mp 296-98 °C; yield 90%; ^1^H NMR (DMSO-*d_6_*): *δ*_H_ 12.26 (s, 1H, NH), 7.48 (d, 1H, *J* = 1.2 Hz, H-7), 7.45 (d, 1H, *J* = 0.9 Hz, H-8), 7.40 (s, 1H, H-5), 6.87 (s, 1H, H-3), 3.33 (s, 3H, COCH_3_), 2.67 (s, 3H, H-6a), 2.26 ppm (s, 3H, H-5a’); ^13^C NMR (DMSO-*d_6_*): *δ*_C_ 193.14 (CO), 160.76 (CO, C-2), 142.57 (C-4), 142.02 (C-5’), 139.16 (C-4’), 138.16 (C-7), 137.42 (C-5), 122.56 (C-8a), 121.14 (C-3), 115.91 (C-6), 115.49 (C-8), 27.67 (CH_3_), 20.38 (6-CH_3_), 9.29 ppm (CH_3_); EI-MS: *m*/*z* 282. *Anal. Calcd for* C_15_H_14_N_4_O_2_: C, 63.82; H, 5.00; N, 19.85. Found: C, 63.71; H, 5.12; N, 20.02.

#### 3.1.3. 4-(4-Acetyl-5-methyl-1*H*-1,2,3-triazol-1-yl)-6-methoxyquinolin-2(1*H*)-one (**3c**)

This compound was found to be a colorless solid; mp 263-65 °C; yield 87%; ^1^H NMR (DMSO-*d_6_*): *δ*_H_ 12.25 (s, 1H, NH), 7.42 (s, 1H, H-5), 7.33 (d, 1H, *J* = 2.1 Hz, H-7), 7.30 (d, 1H, *J* = 2.1 Hz, H-8), 6.90 (s, 1H, H-3), 3.78 (s, 3H, OCH_3_), 3.33 (s, 3H, COCH_3_), 2.47 ppm (s, 3H, H-5a’); ^13^C NMR (DMSO-*d_6_*): *δ*_C_ 193.11 (CO), 160.40 (CO, C-2), 154.73 (C-6), 142.62 (C-4), 141.71 (C-5’), 139.16 (C-4’), 133.99 (C-5), 121.55 (C-8a), 121.17 (C-3), 117.48 (C-4a), 55.54 (OCH_3_), 27.66 (COCH_3_), 9.32 ppm (CH_3_); EI-MS: *m*/*z* 298 (M^+^, 35). *Anal. Calcd for* C_15_H_14_N_4_O_3_: C, 60.40; H, 4.73; N, 18.78. Found: C, 60.51; H, 4.66; N, 18.91.

#### 3.1.4. 4-(4-Acetyl-5-methyl-1*H*-1,2,3-triazol-1-yl)-8-methylquinolin-2(1*H*)-one (**3d**)

This compound was found to be a colorless solid; mp 255-57 °C; yield 71%; ^1^H NMR (DMSO-*d_6_*): *δ*_H_ 12.76 (s, 1H, NH), 7.79 (m, 1H, H-6), 7.25 (d, 2H, *J* = 1.2 Hz, H-5,7), 6.93 (s, 1H, H-3), 3.73 (s, 3H, COCH_3_), 3.45 (s, 3H, N-CH_3_), 2.45 ppm (s, 3H, H-5a’); ^13^C NMR (DMSO-*d_6_*): *δ_C_* 193.12 (CO), 160.55 (CO, C-2), 142.49 (C-4), 141.12 (C-5’), 140.12 (C-4’), 139.60 (C-7), 137.49 (C-5), 123.41 (C-8a), 122.12 (C-6), 120.12 (C-3), 116.41 (C-8), 115.12 (C-4a), 27.64 (CH_3_), 20.31 (N-CH_3_), 9.28 ppm (CH_3_); EI-MS: *m*/*z* 282. *Anal. Calcd for* C_15_H_14_N_4_O_2_: C, 63.82; H, 5.00; N, 19.85. Found: C, 63.70; H, 4.87; N, 19.96.

#### 3.1.5. 4-(4-Acetyl-5-methyl-1*H*-1,2,3-triazol-1-yl)-1-methylquinolin-2(1*H*)-one (**3e**)

This compound was found to be a pale yellow solid; mp 188-90 °C; yield 88%; ^1^H NMR (DMSO-*d_6_*): *δ*_H_ 7.74 (m, 2H, H-6,7), 7.29 (d, 1H, *J* = 6.6 Hz, H-5), 7.25 (d, *J* = 1.2 Hz, 1H, H-8), 6.93 (s, 1H, H-3), 3.72 (s, 3H, N-CH_3_), 3.34 (s, 3H, COCH_3_), 2.45 ppm (s, 3H, H-5a’); ^13^C NMR (DMSO-*d_6_*): *δ*_C_ 193.09 (CO), 160.20 (CO, C-2), 142.56 (C-4), 141.12 (C-5’), 140.12 (C-4’), 139.33 (C-7), 132.49 (C-5), 123.95 (C-8a), 122.91 (C-6), 120.38 (C-3), 116.41 (C-8), 115.60 (C-4a), 29.65 (N-CH_3_), 27.64 (COCH_3_), 9.22 ppm (CH_3_-5a’); EI-MS: *m*/*z* 282. *Anal. Calcd for* C_15_H_14_N_4_O_2_: C, 63.82; H, 5.00; N, 19.85. Found: C, 63.69; H, 5.13; N, 20.01.

#### 3.1.6. 4-(4-Benzoyl-5-phenyl-1*H*-1,2,3-triazol-1-yl)quinolin-2(1*H*)-one (**3f**)

This compound was found to be a pale yellow solid; mp 250-52 °C; yield 82%; ^1^H NMR (DMSO-*d_6_*): *δ*_H_ 12.22 (s, 1H, NH), 8.20-8.17 (m, 2H, Ar-H), 7.71-7.66 (t, 2H, *J* = 6.3 Hz, Ar-H), 7.59-7.55 (t, *J* = 7.8 Hz, 3H, Ar-H), 7.45-7.42 (m, 2H, Ar-H), 7.36-7.32 (m, 3H, Ar-H), 7.16-7.13 (m, 2H, Ar-H), 6.96 ppm (s, 1H, H-3); ^13^C NMR (DMSO-*d_6_*): *δ*_C_ 186.18 (CO), 160.70 (C-2), 143.04 (C-4), 142.65 (C-4’), 142.65, 142.58 (Ph-C), 138.96 (C-5’), 136.88 (C-7), 133.38 (C-6), 132.13 (C-5), 130.27, 130.07, 129.72, 128.41, 128.32, 125.19 (Ar-CH), 123.63 (C-8a), 122.24 (C-3), 115.82 (C-8), 115.64 ppm (C-4a); EI-MS: *m*/*z* 392 (M^+^). *Anal. Calcd for* C_24_H_16_N_4_O2: C, 73.46; H, 4.11; N, 14.28. Found: C, 73.55; H, 4.17; N, 14.14.

#### 3.1.7. 4-(4-Benzoyl-5-phenyl-1*H*-1,2,3-triazol-1-yl)-6-methylquinolin-2(1*H*)-one (**3g**)

This compound was found to be a pale yellow solid; mp 278-80 °C; yield 85%; ^1^H NMR (DMSO-*d_6_*): *δ*_H_ 12.21 (s, 1H, NH), 8.21-8.18 (d, *J* = 8.1 Hz, 2H, Ar-H), 7.71-7.67 (t, *J* = 7.2 Hz, 2H, Ar-H), 7.60-7.55 (t, *J* = 7.5 Hz, 2H, Ar-H), 7.44-7.30 (t, *J* = 6.3 Hz, 3H, Ar-H), 7.35-7.28 (m, 3H, Ar-H), 6.94 (s, 1H, H-5), 6.86 (s, 1H, H-3), 2.26 ppm (s, 3H, CH_3_); ^13^C NMR (DMSO-*d_6_*): *δ*_C_ 186.08 (CO), 160.52 (C-2), 142.70 (C-4), 142.58 (C-4’), 137.03, 136.89 (Ph-C), 133.43 (C-7), 133.32 (C-5), 132.13 (C-7), 130.24 (C-6), 130.01, 129.70, 128.39, 128.24, 125.33 (Ar-CH), 122.84 (C-8a), 121.99 (C-3), 115.73 (C-8), 115.48 (C-4a), 20.33 ppm (CH_3_); EI-MS: *m*/*z* 406 (M^+^, 5). *Anal. Calcd for* C_25_H_18_N_4_O_2_: C, 73.88; H, 4.46; N, 13.78. Found: C, 74.03; H, 4.61; N, 13.90.

#### 3.1.8. 4-(4-Benzoyl-5-phenyl-1*H*-1,2,3-triazol-1-yl)-6-methoxyquinolin-2(1*H*)-one (**3h**)

This compound was found to be a colorless solid; mp 290-92 °C; yield 87%; ^1^H NMR (DMSO-*d_6_*): *δ*_H_ 12.18 (s, 1H, NH), 8.17-8.14 (d, *J* = 7.5 Hz, 2H, Ar-H), 7.71-7.66 (t, *J* = 7.5 Hz, 2H, Ar-H), 7.59-7.54 (t, *J* = 7.5 Hz, 2H, Ar-H), 7.44-7.42 (d, *J* = 7.2 Hz, 2H, Ar-H), 7.36-7.34 (d, *J* = 4.8 Hz, 4H, Ar-H), 7.23 (s, 1H, Ar-H), 6.89 (s, 1H, H-3), 3.89 ppm (s, 3H, OCH_3_); ^13^C NMR (DMSO-*d_6_*): *δ*_C_ 186.44 (CO), 160.46 (C-2), 154.87 (C-6), 142.73 (C-4), 137.06, 133.72 (Ph-C), (C-4’), 137.03, 136.89 (Ph-C), 133.43 (C-7), 133.32 (C-5), 132.13 (C-7), 130.45 (C-6), 130.35, 129.90, 128.61, 128.56, 125.33 (Ar-CH), 122.84 (C-8a), 121.70 (C-3), 117.84 (C-8), 116.18 (C-4a), 55.80 ppm (OCH_3_). EI-MS: *m*/*z* 422 (M^+^). *Anal. Calcd for* C_25_H_18_N_4_O_3_: C, 71.08; H, 4.29; N, 13.26. Found: C, 71.19; H, 4.13; N, 13.41.

#### 3.1.9. 4-(4-Benzoyl-5-phenyl-1*H*-1,2,3-triazol-1-yl)-8-methylquinolin-2(1*H*)-one (**3i**)

This compound was found to be a colorless solid; mp 276-78 °C; yield 77%; ^1^H NMR (DMSO-*d_6_*): *δ*_H_ 11.38 (s, 1H, NH), 8.20-7.01 (m, 13H, Ar-H), 6.96 (s, 1H, H-3), 3.35 ppm (s, 3H, CH_3_); ^13^C NMR (DMSO-*d_6_*): *δ*_C_ 186.14 (CO), 161.05 (CO, C-2), 143.48 (C-4), 142.61 (C-5’), 137.36, 136.87 (Ph-C), 133.35 (C-4’), 130.23 (C-8a), 130.04, 129.70, 128.38, 128.29, 125.18 (Ar-CH), 124.41 (C-5), 122.56 (C-8), 122.02 (C-6), 121.48 (C-3), 115.88 (C-4a), 17.41 ppm (CH_3_); EI-MS: *m*/*z* 406 (M^+^). *Anal. Calcd for* C_25_H_18_N_4_O_2_: C, 73.88; H, 4.46; N, 13.78. Found: C, 73.69; H, 4.55; N, 13.60.

#### 3.1.10. 4-(4-Benzoyl-5-phenyl-1*H*-1,2,3-triazol-1-yl)-1-methylquinolin-2(1*H*)-one (**3j**)

This compound was found to be a pale yellow solid; mp 227-29 °C; yield 80%; ^1^H NMR (DMSO-*d_6_*): *δ*_H_ 8.20-8.18 (d, *J* = 8.1 Hz, 1H, Ar-H), 7.75-7.06 (m, 14H, Ar-H), 6.77 (s, 1H, H-3), 3.65 ppm (s, 1H, N-CH_3_); ^13^C NMR (DMSO-*d_6_*): *δ*_C_ 186.13 (CO), 159.95 (C-2), 142.74 (C-4), 142.62 (C-4′), 141.91, 141.58 (Ph-C), 139.70 (C-5′), 138.48 (C-7), 133.38 (C-6), 132.51 (C-5), 130.25, 130.10, 129.70, 128.99, 128.87, 126.36 (Ar-CH), 124.21 (C-8a), 122.96 (C-3), 116.48 (C-8), 115.54 (C-4a), 29.55 N-CH_3_); EI-MS: *m*/*z* 406 (M^+^). *Anal. Calcd for* C_25_H_18_N_4_O2: C, 73.88; H, 4.46; N, 13.78. Found: C, 73.72; H, 4.61; N, 13.66.

### 3.2. Biochemical Assays

#### 3.2.1. Cell Viability Assay

The human mammary gland epithelial (MCF-10A) cell line was applied to assess the viability of the new derivatives **3a**–**j** [66,67]. Refer to Additional information (Supplementary File).

#### 3.2.2. Antiproliferative Assay

The MTT assay was used to compare the antiproliferative activity of **3a**–**j** to four human cancer cell lines: colon cancer (HT-29) cell line, pancreatic cancer (Panc-1) cell line, lung cancer (A-549) cell line, and breast cancer (MCF-7) cell line, against erlotinib as the reference [68]. Refer to Additional information (Supplementary File).

#### 3.2.3. EGFR Inhibitory Assay

Compounds **3f**, **3g**, **3h**, and **3j**, the most effective antiproliferative derivatives, were evaluated for their inhibitory action on EGFR as a potential target for their antiproliferative action [69]. Refer to Additional information (Supplementary File).

#### 3.2.4. BRAF^V600E^ Inhibitory Assay

Compounds **3f**, **3g**, **3h**, and **3j** were tested for their ability to inhibit mutant BRAF (BRAF^V600E^) according to reported procedures [62]. Refer to Additional information (Supplementary File).

#### 3.2.5. EGFR^T790M^ Inhibitory Assay

Compounds **3g** and **3h** were tested for their efficacy on the mutant EGFR (EGFR ^T790M^) receptor type using the previously reported method [59]. Refer to Additional information (Supplementary File).

#### 3.2.6. Apoptosis Assay

##### Caspase-3 Assay

The most potent derivatives in all in vitro studies, compounds **3f**, **3g**, and **3h**, were tested as caspase-3 activators against human epithelial cancer cell line (A-594) [29]. See Additional information (Supplementary File).

##### Caspase 8, Bax, and Bcl-2 Level Assays

Compounds **3g** and **3h** were investigated further for their effect on caspase-8, Bax, and antiapoptotic Bacl-2 levels against the human epithelial cancer cell line (A-594) using staurosporine as a control [29]. See Additional information (Supplementary File).

#### 3.2.7. Antioxidant Activity

The scavenging of stable free radicals by 2,2-diphenyl-1-picrylhydrazyl (DPPH) was used to investigate the potential antioxidant properties of compounds **3f**, **3g**, and **3h**, using Trolox as a control [75]. See Additional information (Supplementary File).

## 4. Conclusions

Through a 1,3-dipolar cycloaddition approach, a novel series of 4-(1,2,3-triazol-1-yl)quinolin-2(1*H*)-ones (**3a**–**j**) was developed and synthesized in high yields and purity. The structures of all newly synthesized compounds were determined using elemental analysis, mass spectrometry, and NMR spectroscopy. The newly synthesized **3a**–**j** showed potential antiproliferative efficacy against a panel of four cancer cell lines with compounds **3f**–**j** being the most effective antiproliferative agents. Compounds **3f**, **3g**, **3h**, and **3j** were explored as possible multi-target inhibitors of EGFR, BRAF^V600E^, and EGFR^T790M^. Our findings demonstrated that compounds **3g** and **3h** can act as multi-target inhibitors. Furthermore, the apoptotic activity of compounds **3f**, **3g**, and **3h** demonstrated that **3g** and **3h** activate caspase-3, 8, and Bax, as well as down-regulate the antiapoptotic Bcl2, and therefore can be classed as apoptotic inducers. Finally, DPPH radical scavenging activity demonstrated that compounds **3g** and **3h** are potent antiproliferative agents with promising antioxidant activity. After structural modifications, these newly synthesized compounds could constitute a new class of antiproliferative agents with multi-target inhibitory action.

## Figures and Tables

**Table 1:** Optimization conditions.

Base	Et_3_N	NaOH	KOH	K_2_CO_3_	T
**3a**	15	18	20	92%	r.t/st
**3f**	11	13	19	82%	

**Table 2:** Antiproliferative action of compounds **3a**–**j**.

Compd.	Cell Viability %	Antiproliferative Activity IC_50_ ± SEM (nM)				
		A-549	MCF-7	Panc-1	HT-29	Average (GI_50_)
**3a**	90	45 ± 4	48 ± 4	46 ± 4	46 ± 4	46
**3b**	93	64 ± 6	68 ± 6	65 ± 6	64 ± 6	65
**3c**	89	38 ± 3	41 ± 4	38 ± 3	39 ± 3	39
**3d**	94	60 ± 6	65 ± 6	62 ± 6	62 ± 6	62
**3e**	87	54 ± 5	58 ± 5	56 ± 4	58 ± 5	57
**3f**	92	27 ± 2	30 ± 3	27 ± 2	28 ± 2	28
**3g**	89	24 ± 2	28 ± 2	26 ± 2	26 ± 2	26
**3h**	93	21 ± 2	24 ± 3	22 ± 2	22 ± 2	22
**3i**	91	32 ± 3	38 ± 3	35 ± 3	35 ± 3	35
**3j**	90	28 ± 2	34 ± 3	30 ± 3	30 ± 3	31
**Erlotinib**	ND	30 ± 3	40 ± 3	30 ± 3	30 ± 3	33

ND: Not Determined.

**Table 3:** IC_50_ of compounds **3f**, **3g**, **3h**, and **3j** against EGFR, BRAF^V600E^, and EGFR^790M^.

Compd.	EGFR Inhibition IC_50_ ± SEM (nM)	BRAF^V600E^ Inhibition IC_50_ ± SEM (nM)	EGFR^T790M^ Inhibition IC_50_ ± SEM (nM)
**3f**	72 ± 6	86 ± 6	ND
**3g**	64 ± 5	73 ± 6	9.70 ± 0.80
**3h**	57 ± 4	68 ± 5	8.40 ± 0.70
**3j**	79 ± 6	97 ± 7	ND
**Erlotinib**	80 ± 5	60 ± 5	ND
**Osimertinib**	ND	ND	8.00 ± 0.70

ND: Not Determined.

**Table 4:** Caspse-3, caspase-8, Bax, and Bcl-2 levels for compounds **3f**, **3g**, **3h**, and Staurosporine on human epithelial cancer cell line (A-594).

Compd.	Caspase-3		Caspase-8		Bax		Bcl-2	
	Conc (Pg/mL)	Fold Change	Conc (ng/mL)	Fold Change	Conc (Pg/mL)	Fold Change	Conc (ng/mL)	Fold Reduction
**3f**	524 ± 5	8	ND	ND	ND	ND	ND	ND
**3g**	587 ± 5	9	2.17	24	315	36	0.85	6
**3h**	715 ± 6	11	2.35	26	336	37	0.60	9
**Staurosporine**	465 ± 4	7	1.85	21	288	32	1.00	5
**Control**	65	1	0.09	1	9	1	5.00	1

ND: Not Determined.

**Table 5:** Antioxidant activity of compounds **3f**, **3g**, and **3h**.

Antioxidant (DPPH Radical Scavenging Activity %)			
Comp.	100 µM	50 µM	10 µM
**3f**	88.5	76.8	61.3
**3g**	92.4	80.2	70.7
**3h**	96.5	83.9	73.5
**Trolox**	95.2	82.5	77.6

## Data Availability

The data will be available upon request.

## Supplementary Materials

The supporting information can be downloaded at: https://www.mdpi.com/article/10.3390/ijms241713300/s1.
